# Psychopathia Sexualis

**Published:** 1893-07

**Authors:** 


### Book Notices.

PSYCHOPATHIA SEXUALIS, WITH ESPECIAL REFERENCE TO CONTRARY SEXUAL INSTINCT. A Medico-Legal Study. By Dr. R. von Krafft-Ebing, Professor of Psychiatry and Neurology, University of Virginia. Authorized translation of the seventh, enlarged and revised, German edition. By Charles Gilbert Chaddock, M. D., Professor of Mental and Nervous Diseases, Marion-Sims College of Medicine, St. Louis; Fellow of the Chicago Academy of Medicine; Corresponding Member of the Detroit Academy of Medicine: Associate Member of the American Medico-Psychological Association, etc. In one Royal Octavo volume, 436 pages, Extra Cloth, $3.00 net; Sheep $4.00 net. *Sold only by subscription.* Philadelphia. The F. A. Davis Company, Publishers, 1914 and 1916 Cherry street.

But for the medico-legal aspect of the subject considered, and the necessity that often arises in the courts of law for a thorough knowledge of the pathology of the sexual instinct, we very much doubt the propriety of writing a book on so disgusting a subject and of recording the filthy details of cases of sexual perversions. Under existing circumstances, however, a work on this subject will be found of immense value to both lawyers and physicians. We have to consider not merely a matter of taste but one of necessity. Few authors have gone into this territory, and perhaps none of them have gone further than Dr. Krafft-Ebing. The author reports a large number of cases from his own experience. The first two sections of the book are devoted to the consideration of the physiology and psychology of the sexual life, and while the author expresses some opinions somewhat at variance with the generally accepted views on these subjects, he brings forward some strong proof, and draws from his own rich experience, in support of his position. He gives a careful description of sedism, perversion, contrary sexual instinct, masochism, fetichism, urnings, etc. A number of cases are reported under these heads. This part of the book will be of vast assistance to the physician in making a proper classification of cases of sexual perversion. The author reports some cases cured by hypnotic suggestions and he attaches much importance to this method of treatment. The book is written in a clear, forcible style, will be easily understood, as both the author and the translator have done their work in a masterly way. We recommend the book to any physician or lawyer who desires to study this subject. H.

